# U-EXCEL’S “@ HOME” PROGRAM: STAFF SUPPORTED BALANCE EXERCISE INTERVENTION AND ADHERENCE FOR LIFE CARE RESIDENTS

**DOI:** 10.1093/geroni/igz038.3533

**Published:** 2019-11-08

**Authors:** Marilyn R Gugliucci, Erica Robertson, Ashley Cronkright, Sujaay Jagannathan

**Affiliations:** 1 University of New England College of Osteopathic Medicine, Biddeford, Maine, United States; 2 University of New England College of Osteopathic Medicine, Maine, United States

## Abstract

Introduction: The University of New England College of Osteopathic Medicine U-ExCEL Program was established in 2006 and specializes in older adult fitness and balance programming. Falls account for $54 billion costs in direct and Indirect costs. Methods: This randomized control single blinded pilot project included an 8 week intervention to measure the impact of supported consistent individual balance programming in individuals’ apartments for a select group of older adults residing in a life care living environment. Twenty residents (75-92 y/o) were recruited, however 12 participants (6 intervention/6 control group) participated in the study. The remaining 8 participants were pulled from the wait list as attrition occurred. Demographic data collection and 6 validated assessments were conducted at baseline and at study completion. The intervention group conducted the Balancing Act (Falls Prevention) Program 3 times/week with social support. The control group only received social support. Data analysis included descriptive statistics, SAS 5.1 was used for non-parametric Mann-Whitney U Test (Wilcoxon Rank Sum Test); a repeated measures ANOVA was also conducted. Results: The effects of the intervention (Balancing Act Program) on Oxygen Saturation (p=0.009), Wong Baker Score (p=0.008), and the Rapid Assessment of Physical Activity (RAPA) 2 (p=0.008) test were statistically significant. The effect of the intervention on all other variables was not statistically significant including validated balance measures. Conclusion: Quantitative measures failed so show significant improvement in balance from the start to the end of the intervention; however improvements were experienced and expressed by the intervention group. Social Support is necessary for adherence.

